# Genomic multiple sequence alignments: refinement using a genetic algorithm

**DOI:** 10.1186/1471-2105-6-200

**Published:** 2005-08-08

**Authors:** Chunlin Wang, Elliot J Lefkowitz

**Affiliations:** 1 Department of Microbiology, University of Alabama at Birmingham, Birmingham, Alabama 35294-2170, USA

## Abstract

**Background:**

Genomic sequence data cannot be fully appreciated in isolation. Comparative genomics – the practice of comparing genomic sequences from different species – plays an increasingly important role in understanding the genotypic differences between species that result in phenotypic differences as well as in revealing patterns of evolutionary relationships. One of the major challenges in comparative genomics is producing a high-quality alignment between two or more related genomic sequences. In recent years, a number of tools have been developed for aligning large genomic sequences. Most utilize heuristic strategies to identify a series of strong sequence similarities, which are then used as anchors to align the regions between the anchor points. The resulting alignment is globally correct, but in many cases is suboptimal locally. We describe a new program, GenAlignRefine, which improves the overall quality of global multiple alignments by using a genetic algorithm to improve local regions of alignment. Regions of low quality are identified, realigned using the program T-Coffee, and then refined using a genetic algorithm. Because a better COFFEE (Consistency based Objective Function For alignmEnt Evaluation) score generally reflects greater alignment quality, the algorithm searches for an alignment that yields a better COFFEE score. To improve the intrinsic slowness of the genetic algorithm, GenAlignRefine was implemented as a parallel, cluster-based program.

**Results:**

We tested the GenAlignRefine algorithm by running it on a Linux cluster to refine sequences from a simulation, as well as refine a multiple alignment of 15 Orthopoxvirus genomic sequences approximately 260,000 nucleotides in length that initially had been aligned by Multi-LAGAN. It took approximately 150 minutes for a 40-processor Linux cluster to optimize some 200 fuzzy (poorly aligned) regions of the orthopoxvirus alignment. Overall sequence identity increased only slightly; but significantly, this occurred at the same time that the overall alignment length decreased – through the removal of gaps – by approximately 200 gapped regions representing roughly 1,300 gaps.

**Conclusion:**

We have implemented a genetic algorithm in parallel mode to optimize multiple genomic sequence alignments initially generated by various alignment tools. Benchmarking experiments showed that the refinement algorithm improved genomic sequence alignments within a reasonable period of time.

## Background

One of the primary goals in analyzing complete genomes is to identify all of the functional regions in the sequences, including genes and regulatory regions. However, this interpretive work is not keeping pace with the avalanche of raw sequence data. This disparity is due in part to the fact that algorithm development for genomic annotation has been relatively slow, and annotation of completely sequenced genomes inevitably depends on human expert knowledge. The most effective method to understand genomic content is to compare multiple genomes of various phylogenetic distances. The coding regions of a large set of common genes can be identified by comparing genomic sequences that are distantly related phylogenetically. In addition, comparing the genomic sequences of divergent non-coding regions that show some degree of conservation can yield important information related to regulation of gene expression, structural organization of the genome, and possibly other yet unknown functions [1]. Finally, functional and evolutionary inferences can be made from comparative genomic analysis. For example, orthologous relationships can suggest the function of a genetic sequence when the function of a similar sequence in another species is known.

One of the major challenges when comparing two or more genomic sequences is producing a high-quality multiple sequence alignment of all the genomes. In recent years, a number of computer programs have been developed for the alignment of large genomic sequences including CHAOS/DIALIGN [2], MUMmer [3], WABA [4], VISTA [5], BLASTZ [6], MAVID [7], and Multi-LAGAN [8]. In general, these tools utilize heuristic algorithms that provide an approximate solution to the problem of generating multiple sequence alignments. These heuristic tools are based on many different paradigms, but they all fall into the "anchor-extension" strategy, wherein a series of strong sequence similarities (*anchors*) are identified first and gaps are filled in (*extension*) by aligning the regions between the anchor points. The resulting alignment is globally correct, but in many cases is suboptimal locally.

In this paper, we describe the development of a program, GenAlignRefine, which improves the overall quality of global multiple sequence alignments by using a genetic algorithm to improve alignments in local regions.

## Implementation

Multiple sequence alignment (MSA) is one of the most difficult problems in computational biology and there are only approximate solutions for all but the smallest alignments [9]. Therefore a number of novel heuristic algorithms have been proposed [10]. There are at least two distinct technical problems remaining: the choice of an objective function (OF) that assesses the quality of an alignment, and the design of an appropriate algorithm to optimize the score from that objective function. Ideally, a biologically meaningful alignment should produce a better OF score than a suboptimal alignment. One popular OF is the sum-of-pair (SP) function, which is a direct extension of the scoring method used in pair-wise alignments [11]. The SP score for an aligned column in the MSA is computed by scoring all of the pair-wise comparisons between each residue in each column of an alignment and adding the scores together. The major shortcoming of the SP function is that the alignment quality is extremely dependent on the choice of a score matrix and gap penalties. In general, this choice is problem-specific. For instance, for closely related input sequences, it might be better to use a score matrix reflecting a short phylogenetic distance. On the other hand, for divergent sequences, it might be more appropriate to choose a score matrix reflecting a longer phylogenetic distance.

One alternative to using the SP function is the COFFEE [12] function, which evaluates the consistency between a multiple alignment and libraries of optimal pair-wise alignments of the same sequences. Although COFFEE does not completely overcome the problems related to the choice of score matrix and gap penalties, it can reduce them. (There are some algorithms such as Dialign, in which the OF does not consider gapped regions. Instead, the OF of Dialign uses the sum of weights of gap free segment pairs [13]). The optimal pair-wise alignment is inevitably affected by the choice of score matrix and gap penalty, but a correct choice will be more likely since it is possible to choose different score matricies and gap penalties adaptively based on the distance detected during the pair-wise alignment process. The COFFEE OF has been shown to be more robust and to lead to better alignments [14]. In our application, we chose to utilize the COFFEE OF as a measure of the optimization of the multiple sequence alignment. Our COFFEE score is calculated based on the following formula:



where N is the number of sequences to be aligned; W_ij _is the percent identity between sequence i and j in the optimal pair-wise alignment library; C_ij _is the number of aligned character pairs that are shared between the multiple alignment and the optimal pair-wise alignment; and L_ij _is the length of the optimal pair-wise alignment of sequences i and j. The optimal pair-wise alignment library is constructed by aligning every pair of input sequences with an implementation of the Needleman-Wunsch algorithm [15].

To optimize an alignment by attempting to maximize its COFFEE score, we chose to implement a genetic algorithm. A genetic algorithm is a stochastic search method based on the concept of biological evolution; i.e., in simulating an evolutionary process in a population of potential solutions, a better solution will evolve [16]. Biological terms are used to describe the evolutionary process. Each potential solution is called a chromosome; a set of chromosomes refers to a population; and successive populations are called generations. To create new chromosomes (or offspring), two types of operators are generally used: mutation, which changes a single chromosome, and crossover, which exchanges information from two or more chromosomes. Based on Darwin's principle of survival of the fittest, chromosomes that perform well on certain fitness functions will have a greater likelihood of producing more offspring. Since the best performing individual in each generation is always selected for the next generation, the solutions in each generation are at least as good as those provided in previous runs. In this way, the genetic algorithm is able to optimize solutions from any source. Using genetic algorithms to solve MSA problems is not a new idea. SAGA [17] successfully applies a genetic algorithm to MSAs by attempting to optimize the weighted sum-of-pairs with natural or quasi-natural affinity gap penalties. Further attempts based on SAGA include SAGA-COFFEE, which tries to optimize the consistency-based objective function [14]. Our approach differs from SAGA in several ways. First, GenAlignRefine is designed to optimize multiple sequence alignments without regards to their length. SAGA is not optimized to align genome-length sequences. Second, in order to reduce the number of genetic operators that need to be utilized, GenAlignRefine pre-aligns each fuzzy region using T-Coffee. This allows us to optimize the application of the genetic operators by using a combination of only 3 operators rather than the full set of 22 operators used in SAGA (see below).

There are two considerations when designing a genetic algorithm. The first is how quickly a genetic algorithm can converge to an optimal solution. The second is the risk of misguiding an optimization process to a solution that appears to be optimal, but in fact resulted from convergence to a local optimum. Genetic algorithms are known to be extremely slow, with some MSA implementations being hundreds of times slower than ClustalW [18,19]. Genomic sequences may be megabases in length, and depending on the similarities between the sequences to be aligned, there may be thousands of poorly aligned regions. For that reason speed is a critical factor. To improve the overall performance of this application, we implemented the entire optimization process as a parallel, cluster-based program.

Based on manual inspection of the multiple genome alignments produced by various tools, we have found that regions encompassing and surrounding gaps are where most of the discrepancies between alignment methods occur. In this study, we concentrated our attention on these "fuzzy" regions. Fuzzy regions were defined as columns in an alignment that contain a gap adjacent to a gap-free region of at least 20 nucleotides. The gap-free regions of 20 nucleotides in length provided a constrained space in the multiple alignment that allowed the refining algorithm to place gaps between the constrained positions. Experimenting with different lengths of gap-free regions showed that a length of 20 was sufficient to allow for reasonable constraint. Longer gap-free regions did not increase the quality of final alignment, but did increase the length of time required for the optimization process.

GenAlignRefine was developed based on the assumption that the original starting alignment is globally correct. With this rationale, the overall genome alignment is shaped by those anchor regions that show strong similarity and are therefore obvious orthologs. These optimal anchor regions are kept intact. Optimization of the fuzzy regions between these anchor points will therefore not reshape the overall alignment, but will improve the overall quality of the alignment by improving each individual local region. One possible reason that the starting alignment might not be globally correct would be if one or more genomes contained large sequence rearrangements in comparison to the other genomes. Regions containing such rearrangements would need to be removed from the analysis since these regions will not be directly alignable. GenAlignRefine handles this problem by removing from consideration fuzzy regions longer than 1000 bases that may contain, in addition to rearrangements, large numbers of repeat sequences. Manual inspection of whole-genome pair-wise dotplots [3,20] were also used to identify these unalignable regions.

Using a Linux cluster in master-slave mode, fuzzy regions from an initial rough global MSA were identified. These fuzzy regions were then sent by the master to one of the slave nodes where a genetic algorithm (described below) was used to optimize the fuzzy region. The process continued until all fuzzy regions were optimized.

**Figure 1:**
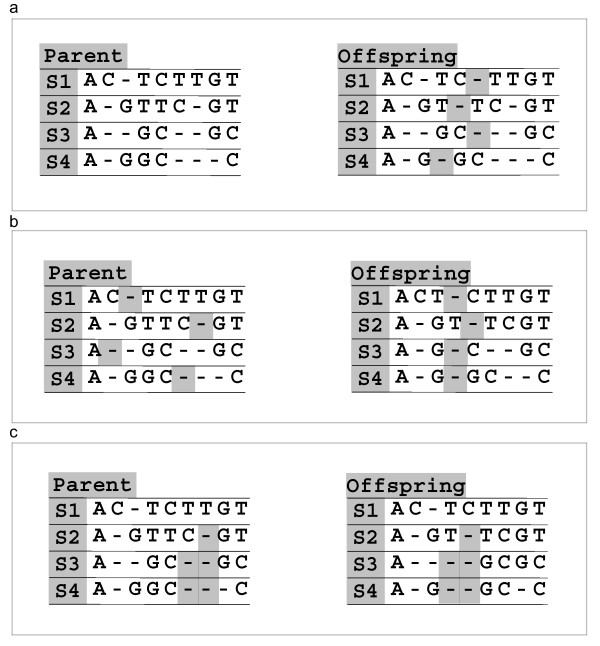
**Mutation operators used in GenAlignRefine**. a) Random_gap operator. Random gaps (shaded hypens) are inserted into the parent alignment to produce the offspring sequences. b) Local_gap_shuffle operator. Gaps in the parent are randomly moved to produce new offspring. c) Block_gap_shuffle operator. Contiguous blocks of gaps are randomly moved to new positions.

It is important for the efficiency of the search process to utilize a reasonable alignment for each fuzzy region as the starting point for optimization. This avoids local optima and yields better results in shorter periods of time. Therefore, in GenAlignRefine, we first used T-Coffee [14] on each of the fuzzy regions to produce an initial alignment, which is then used as the starting point for the genetic algorithm, thereby becoming the first chromosome from which successive generations originate. This strategy has been proven successful in other studies [21] and we have found it to be very efficient. Furthermore, since each potential solution in a population is derived from the T-Coffee alignment, the simplest way to optimize the COFFEE score is to re-arrange gaps in the alignment. For that reason, we implemented a subset of the mutation operators from SAGA [17] that perform gap re-arrangement: random_gap, local_gap_shuffle, and block_gap_shuffle (see Fig. 1). The random_gap operator randomly inserts a gap into every sequence of the alignment. The local_gap_shuffle operator shuffles one gap in every sequence by randomly moving it to a different position in that sequence. The block_gap_shuffle randomly moves gaps in selected columns of every sequence of the alignment to different positions in that alignment. In addition, any columns containing only gaps are deleted in each generation. A combination of random_gap, local_gap-shuffle, and block_gap_shuffle was able to effectively simulate any single mutation operator in SAGA. Previous studies have shown that operator schedules (setting a probability and frequency for use of each genetic operator) did not improve the performance of SAGA compared to the uniform selection of SAGA operators [21]. After experimenting with different operator scheduling strategies, we chose to use the three operators at equal frequency.

In summary, the overall process used to generate refined multiple sequence alignments starts with a set of sequences that are initially aligned using a genome sequence MSA tool such as Multi-LAGAN. Fuzzy regions within these alignments are identified and individually realigned using T-Coffee. These realigned regions are then refined using our implementation of the indicated set of genetic algorithm operators. All computation following generation of the initial multiple sequence alignment takes place on a Linux cluster.

GenAlignRefine was implemented in Perl to take advantage of the convenience in manipulation of biological sequences provided by the Bioperl [22] application programming interface (API). To bridge the gap between Perl and the message passing interface (MPI) [23] API which is implemented in C/C++ and is required for our parallel implementation, we also provide a wrapper module that ports to Perl only those MPI C/C++ procedures necessary for this application. The implementation of an efficient Needleman-Wunsch algorithm [15] coded in C/C++ and ported to PERL was used to construct pair-wise alignment libraries.

## Results and discussion

We performed our benchmarking experiment on a 32-node Linux cluster in the Department of Computer and Information Science at the University of Alabama at Birmingham. All machines have 1.6 GHz Dual AMD Opteron™ Processors, 2 GB of RAM, and are connected via Gigabit Ethernet.

The lack of a "gold standard" for assessment of multiple sequence genome alignments makes it difficult to assess the performance of multiple genome alignment tools. In this study, we chose a simulation-based approach to benchmark the results produced by GenAlignRefine. Multiple sequences along with the correct, "optimal" multiple alignment of these sequences as generated by the software tool Rose (random model of sequence evolution) [24] have been widely used to benchmark the performance of multiple alignment tools [25] and phylogenetic analyses [26]. We started with a sequence alignment created by Rose which contains 9 sequences comprising about 100,000 nucleotides each. Sequence generation began with 1 randomly generated ancestral sequence composed of equal nucleotide base frequencies. From that sequence, the 9 test sequences were generated based on the HKY evolutionary model of point substitution [27] using a transition/transversion bias of 2.5. The insertion and deletion threshold was set to allow insertions and/or deletions 5% of the time. The mean base substitution rate was set to 0.05 substitutions per site. The tree was set to ((a:.2,b:.5):.1,(c:.4,d:.5,e:.4):.2,(f:.3,g:.4):.3,(h:.4,i:.5):.1). For each sequence, the mutation probability of each nucleotide position was set to either 0.0, 0.3, 0.6, 0.9, or 1. Using this set of 9 sequences, we generated two new alignments using the programs Multi-LAGAN [8] which was run locally using the default options, and CHAOS/DIALIGN [2] which was run using the available web application [28]. Each of the two new alignments was then subjected to refinement by GenAlignRefine. We then measured the consistencies between the alignments by comparing each of the four new alignments to the original simulated alignment produced by Rose. The consistency between any two alignments, A and B is defined as the ratio between the number of identical character pairs between the two alignments, and the total number of character pairs in alignment B. The results from this comparison are provided in table 1. In each case, GenAlignRefine was able to improve the quality of each multiple genome sequence alignment by at least 7% as measured by an increase in the number of pair-wise matches to the "optimal" alignment as constructed by Rose. It was also apparent that the quality of the final alignment was dependent on the quality of the original alignment prior to refinement.

**Table 1:** Performance of GenAlignRefine on simulated data.

Program	Before Refinement	After Refinement
CHAOS/DIALIGN	78.0%*	85.1%
Multi-LAGAN	86.3%	93.0%

* The numbers indicate the consistency between the alignment generated with the genome alignment tool and the "correct" alignment generated by Rose (see text).

In addition to the above benchmarking experiments, we also conducted a study to demonstrate the usefulness of the program GenAlignRefine by refining the genome alignment of complete genome sequences available for the virus genus, *Orthopoxvirus *that includes variola virus, the agent responsible for causing smallpox. Genome sequences used in this analysis were obtained mostly from GenBank and included vaccinia virus strains Copenhagen [GenBank: M35027], Western Reserve (WR) [GenBank: AY243312], and Tian Tan [GenBank: AF095689] (with updates from Dr. Chris Upton of the University of Victoria); variola major virus strains Bangladesh [GenBank: L2579] and India [GenBank: X69198]; variola minor virus strain Garcia [GenBank: Y16780]; camelpox virus strains CMS [GenBank: AY009089] and M-96 [GenBank: AF438165]; cowpox virus strains Brighton Red [GenBank: AF482758] and GRI-90 [GenBank: X94355]; ectromelia virus strain Moscow [GenBank: AF012825]; and monkeypox virus strain Zaire [GenBank: AF380138]. Genome sequences of monkeypox virus strain WRAIR 7–61 [GenBank: AY603973] and rabbitpox virus strain Utrecht [GenBank: NC_005858] were kindly provided by Dr. Mark Buller of Saint Louis University. The genome sequence of ectromelia virus strain Naval was obtained from [29]. An initial alignment of these 15 *Orthopoxvirus *genomic sequences was created using Multi-LAGAN [8]. The overall length of the alignment was approximately 260,000 nucleotides.

Two considerations in implementing our genetic algorithm were when to stop the optimization process and how to breed the next generation. In our benchmarking experiment, if there was no improvement in an alignment's COFFEE score for 500 generations, the optimization process was stopped and the alignment with the best COFFEE score was returned to the master node. As the population size increases, the risk of falling into a local, suboptimal alignment decreases, but so does the speed of the optimization process. Since our starting alignments are derived from T-Coffee, it is assumed that they are close to the global optimum. For that reason, and for the sake of efficiency, we try to keep the population size relatively small. From each pool of 1000 individuals (alignments generated by application of the genetic algorithm), only the top 100 individuals with the best COFFEE scores are allowed to breed. In addition, we use an elite selection strategy in which some of the fittest individuals from the first generation are allowed to carry over unaltered into the second generation. We also permit individuals having the best COFFEE scores to have more offspring. All of these parameters are adjustable.

**Figure 2:**
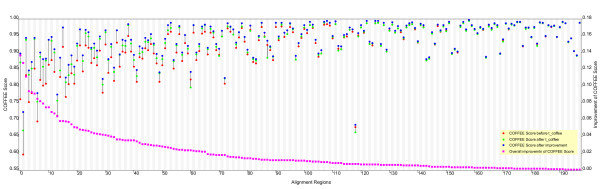
**Improvement of COFFEE score for fuzzy regions**. The 200 fuzzy regions derived from the starting Orthopoxvirus alignment that showed improvement following application of GenAlignRefine are displayed. For clarity, regions are sorted according to the overall improvement in COFFEE score. Vertical bars connect dots that show the improvement in COFFEE score for each region at each step in the refinement process. Red dots plot the original COFFEE score of the Multi-LAGAN-generated alignment for each region; green dots plot the COFFEE score of the same region after realignment by T-Coffee; blue dots indicate the COFFEE score of the same region after optimization by the genetic algorithm. The small magenta squares plot the overall improvement in COFFEE score for each region.

In general, regions with gaps are most likely to be discordant and therefore in need of improvement. However, even for closely related species, not all regions in their genomic sequences can be aligned. For instance, the regions at the ends of poxvirus genome sequences contain variable numbers of repeat sequences and some of these repeating units are species-specific [30] and thus cannot be aligned. In our study, fuzzy regions longer than 1000 bases were considered unalignable, so these regions were not subject to optimization. In our orthopoxvirus alignment, there were 18 fuzzy regions longer than 1000 bases, all of which occurred at the ends of the original alignment. Between these unalignable genomic regions, there were about 400 gapped (fuzzy) regions that were then subjected to the genetic algorithm. Using 40 processors, it took 150 minutes to optimize all of these regions. Only some 200 regions were actually improved by the genetic algorithm with the remaining 200 regions already showing optimal COFFEE scores. Figure 2 displays the improvement in the 200 fuzzy regions based on the COFFEE scores. It is apparent that, in general, regions with lower initial COFFEE scores showed more improvement while regions with higher initial COFFEE scores showed less improvement.

An improvement in COFFEE score is only one possible measure that might reflect an actual improvement in alignment quality. And since for these poxvirus sequences, there is no "correct" alignment for comparison as there was for the simulation, we chose to measure improvement by simply demonstrating an increase in the overall percentage identity calculated between all pair-wise sequence comparisons, along with a decrease in the length of the overall alignment due to the introduction of fewer gaps. An increase in the percentage identity can be achieved by simply inserting greater numbers of gaps into the alignment without necessarily improving the overall quality of the alignment. However, when the overall percentage identity is increased concomitant with a decrease in the length of the alignment, then in general, we would argue that the overall quality of the alignment has been improved. The alignment of the 15 Orthopoxvirus genomic sequences produced by Multi-LAGAN was 259817 nucleotides in length and showed 96.0% identity [see Additional file 1]. After refinement by GenAlignRefine, the length of the alignment was 258593 with 96.2% identity [see Additional file 2]. The improvement in the percentage identity was marginal for the overall alignment, but for each fuzzy region, the improvement was much higher. Significantly, this occurred at the same time that the overall alignment length decreased – through the removal of gaps – by approximately 200 gapped regions representing roughly 1,300 gaps. Therefore, the increase in the overall alignment quality was substantial.

The three mutation operators used in the genetic algorithm were effective after using T-Coffee to initially realign each fuzzy region of the starting alignment thus producing a seed for subsequent improvement. This is similar to what has been seen in previous studies [21]. As the resulting alignment from T-Coffee can be viewed as an approximation to the optimal result, starting with this alignment, which is presumably close to the optimal result in the multiple alignment search space, should decrease the chance of becoming trapped into a local optimum. And although there is still a risk that the optimization process will be misguided to a local optimum, the chance of this occurring should be small.

Genetic algorithms are known to be slow and computationally intensive compared to other methods [19]. However by using appropriate design parameters along with a large computing cluster, we have shown that GenAlignRefine can be used to efficiently and effectively improve multiple sequence alignments of whole genome sequences.

## Availability and requirements

GenAlignRefine is freely available under the Artistic License described by the Open Source Initiative [31]. The source code can be downloaded via ftp [32]. Contact elliotl@uab.edu for information on obtaining the software. It has been tested on an AMD Opteron™ Processor-based Linux cluster with LAM/MPI and should be compatible with other implementations of MPI.

## Authors' contributions

CW was responsible for the conception, design, implementation, and testing of the GenAlignRefine. EJL contributed to its conception and testing and provided overall project coordination. Both authors have read and approved the final manuscript.

## Supplementary Material

Additional File 1 Orthopoxvirus multiple genome alignment prior to optimization. Original Orthopoxvirus alignment produced by Multi-LAGAN in Clustal .aln format. Click here for file

Additional File 2 Orthopoxvirus multiple genome alignment after optimization. Final Orthopoxvirus alignment following improvement by GenAlignRefine in Clustal .aln format. Click here for file
